# Adaptive focal lengths in white light focusing Fresnel lenses enabled by reflective-type and phase-only spatial light modulator

**DOI:** 10.1038/s41598-023-44231-2

**Published:** 2023-10-09

**Authors:** Pravinraj Selvaraj, Sheng-Le Wang, Tsung-Yi Hou, Cheng-Kai Liu, Ko-Ting Cheng

**Affiliations:** Department of Optics and Photonics, National Central University, Taoyuan, 320317 Taiwan

**Keywords:** Optics and photonics, Optical techniques

## Abstract

Fresnel zone plates (FZPs) are widely used in integrated optical systems to meet new cutting-edge demands for photonic integration and device miniaturizing. However, their use in applications of cross-scale fabrication still faces several obstacles, such as low efficiency, fixed focal length, single wavelength, large size, and complicated fabrication. Here, we first examine a novel adaptive focal length in white light focusing by using reflective-type and phase-only spatial light modulator (RLC-SLM) based on a liquid crystal on silicon. The device achieves a maximum diffraction efficiency of approximately 38% at primary focal points of binary phase-type FZPs throughout the visible range (red, green, and blue wavelengths). The RLC-SLM focuses the light of the desired wavelength while other sources are defocused. White light focusing and color separation are demonstrated by sequentially and additively switching different FZPs. These recent advances show that optically tunable FRZs are promising potential candidates to enhance adaptive camera systems, microscopes, holograms, and portable and wearable devices, thereby opening up novel possibilities in optical communications and sensing.

## Introduction

Over the past decades, Fresnel lenses have emerged as a key player in photonics, 3D displays, optical imaging, diffractive networks, high-dynamic all-focus imaging, and long-distance optical communication^1–7^. However, conventional Fresnel lenses are fabricated by electron-beam writing^8^, which has several drawbacks, such as these lenses often suffer from low diffraction efficiency due to electron-beam precision and speed limitations, especially for complex or large apertures. The focal length is fixed during fabrication, necessitating a complete redesign for any changes. These lenses are tailored for a single wavelength, restricting their adaptability for broad or multi-wavelength applications. Moreover, the fabrication process is complex and time-consuming, requiring specialized equipment and various steps. Similarly, thin-film deposition also suffers from lower diffraction efficiency^9^, particularly at shorter wavelengths, due to defects in the deposited layers. Consequently, switching the focal length without re-fabricating makes them challenging to use in multi-wavelength scenarios. This process involves precise control over layer thickness and composition^9^. Liquid crystals (LCs) are promising candidates for fabricating switchable LC Fresnel zone plates (FZP). The FZP is a typical example of a diffractive optical device composed of alternately transparent and opaque concentric rings or zones of different thicknesses (phase type)^10^ or optical transmittances (amplitude type)^11^. Since the light beams transmitted or reflected by FZPs interfere beneficially at the intended points, they are frequently used in optical systems as the refractive lens for focusing and have been widely used in optical tweezers, microsensors, and interconnections^12^. Given their excellent electro-optical characteristics and low operating voltage, LCs have been used for displays, light shutters, polarization converters, lenses, and other electrically switchable devices^13–19^. In the diffraction efficiency, LC FZP can be controlled by varying electrical fields due to the orientations as refractive indices of LCs in the odd and even zones that can be modulated, which causes the phase difference of light.

To develop electrically switchable LC FZP, scholars have established numerous methods, including UV-modified alignment films^20,21^, patterned electrodes^20^, polymer and blue phase LC composite (PBPLC)^22^, polymer network LCs (PNLCs)^23,24^, polymer-dispersed LCs (PDLCs)^25^, and photoaligned dye-doped LCs (DDLCs)^26^. The development of PDLC and PNLC devices is straightforward, but they consistently exhibit high light scattering behavior, lowering the FZPs’ resolving power. The DDLC FZP is fabricated by photo-induced dye absorption, which requires a powerful laser (40 mW/cm^2^) to pump azo dyes into LC alignment. Moreover, the shortages of methyl red adsorption include instability under sunlight, red color tint, and thermal instability^26^. The PBPLC Fresnel lens operates at an extremely high voltage of 200 V, which leads to electrode breakage and considerable hysteresis effect. High exposure energies are also necessary for the UV-modified alignment films’ LC FZP profile generation, and the switching OFF time (180 ms) is similarly long. However, the abovementioned LC Fresnel lenses exhibit common disadvantages, such as high voltage operation, light scattering, long switching times, limitations of a single wavelength, fixed focal length, and hampering their practicality.

In this study, we demonstrate white light-focusing Fresnel lenses with adjustable focal lengths by using a reflective-type and phase-only spatial light modulator (SLM) to address the previous shortcomings. SLMs are essential components in various applications because they can spatially control light amplitude, phase, or polarization, including developing holographic and AR/VR displays, bio-imaging, and quantum computing^27–30^. SLMs based on LC on silicon (LCoS) are widely known for producing high-resolution and rapid switching modulations of phase and amplitude of light with individual electrically addressable pixels^27^. Moreover, Reflective-type SLMs, including LCoS devices, are essential tools widely employed in various optical applications due to their ability to control light’s spatial properties, such as amplitudes and phases. These devices are particularly well-suited for applications requiring rapid and high-resolution light modulation, such as display applications, optical tweezers, holographic projection, and Imaging and projection. The implication is that the initial phase of incoming light may be modified by controlling the spatial distribution of phases for each pixel. The phase-only reflective type LC-SLM modulates the effective phase of each pixel by adjusting its grayscales^27^. As such, the phase difference between the two neighboring ring sections of each FZP can be electrically adjusted to be π close to the theoretical maximum focusing efficiency (first-order diffraction efficiency) value of 40.5%, which is greater than the 25.6% of binary amplitude FZPs^31,32^. Thus far, however, these RLC-SLM-based FZPs devices and their extension to color separation and additive color approaches have not been addressed.

In this study, we introduce and examine a novel adaptive focal length in white light-focusing by using an RLC-SLM based on LCoS. The RLC-SLM device achieves maximum diffraction efficiency at the primary focal point across the visible light source. On this basis, we use FZPs to extract specific colors, which effectively split the broadband light source at their respective focus points. This method enables precise focusing of light with the desired wavelength, avoiding issues of defocus or misalignment. Furthermore, we successfully demonstrate white light focusing by using color sequential and additive color approaches with real-time RLC-SLM switches controlled by FZPs. The developed FZPs have a wide operating range in the visible spectrum, and are thus highly suitable for adaptive camera systems, portable devices, and communication applications.

## Experimental setup

**Figure 1:**
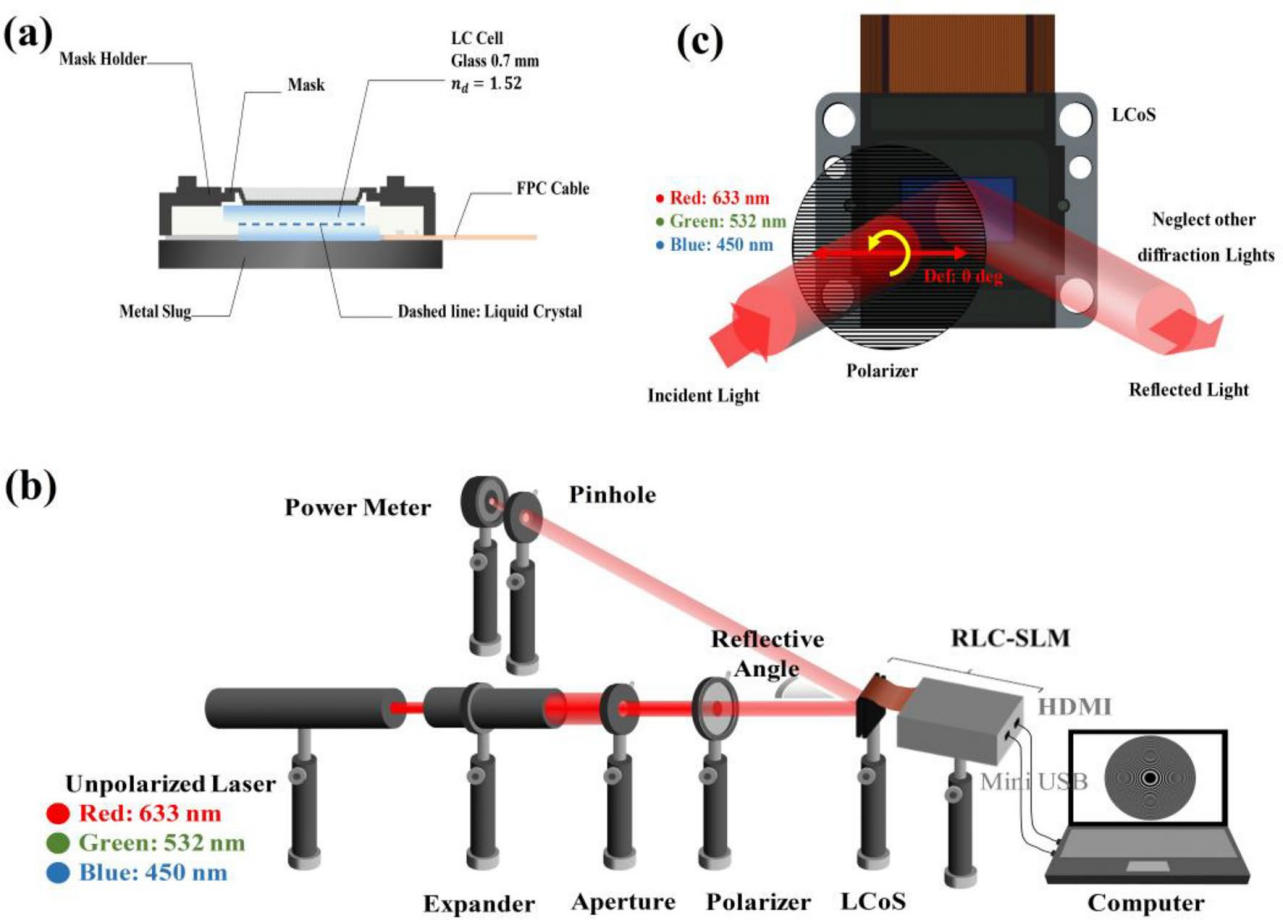
LCoS-based RLC-SLM: (**a**) schematic diagram; (**b**) experimental setup for analyzing the diffraction efficiencies of FZPs; (**c**) Schematics and the input and output light beams.

Figure 1a shows a schematic of the LCoS-based RLC-SLM. The adopted micro-display for phase modulation with good pixel quality includes the following specifications: LC alignment, diagonal length of the active area, resolution, aperture ratio, grayscales, pixel pitch, and response time for vertically aligned LC, 0.55″, 1920 × 1080, ~ 93%, 128, 6.4 μm, and < 30 ms, respectively. Figure 1b shows the experimental configuration that is used to examine the focusing efficiency across multiple FZPs based on RLC-SLM. This study uses the phase-only reflective LC-SLM type JD8554 from Jasper Display Corporation. To examine the focusing efficiencies, we also use un-polarized lasers—such as red (R, λ_R_ = 632.8 nm), green (G, λ_G_ = 532 nm), and blue (B, λ_B_ = 450 nm)—as the light sources. To create a uniform light spot, we collimated incoming light by passing it through a beam expander and an aperture. In RLC-SLM, the polarizer’s transmissive axis is arranged to be parallel to the rotation direction of the LCs, and thereby ensuring that the phase modulations produced by the LCs did not cause any phase retardation, which can alter the output light polarization. According to Fig. 1c, such a direction is determined as 0° of *θ*. A 10° angle is also created between the input and output light beams to facilitate evaluation. A photo-detector then measures the optical strengths of the input and output light beams to determine the focusing efficiencies.

As discussed above, RLC-SLM can be used to illustrate the white light focusing, color separation focusing, modification of the colors of focused points, and adjustable focusing efficiency based on color sequential and additive color approaches. Therefore, swapping out the numerous optical components in Fig. 1b is necessary to complete the experiments of the observations of diffraction patterns and their spectra for various colors of light sources. In place of the photo-detector and the aperture (output side), the replacements consist of the following: (i) an observation screen; (ii) a white light source; and (iii) a spectrum meter. Three identically-intensity laser (RGB) light beams are used as the artificial white light source and the incident one may be focused separately on the desired focal plane by the appropriate FZPs shown on the LC-SLM. Thus, this part demonstrates the separation and focus of the white light’s various colors. By quickly switching the FZPs, the combined colors of focused light can be shown due to the additive color approach and the real-time switchable spatial phase modulation with a quick response of the LC-SLM. Then, the human visual system combines the sequential focused colors to create the combined colors, including white, cyan, magenta, yellow, and others.

## Results and discussion

**Figure 2:**
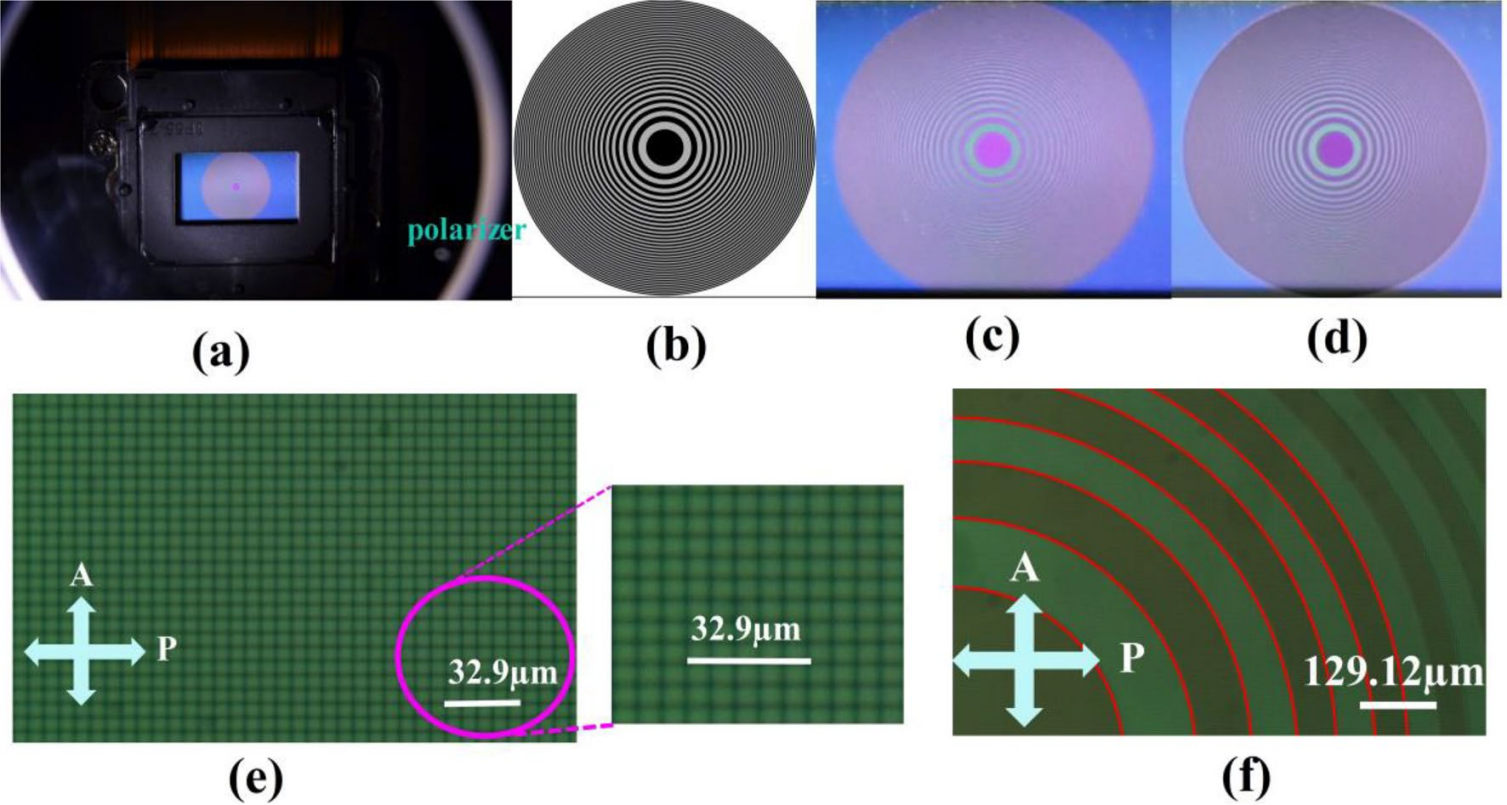
(**a**) Observation of FZP on LCoS through a linear polarizer; (**b**) theoretical illustration of FZP plotted using Matlab software, depicting the spatial information; (**c**) presentation of FZP on LCoS; (**d**) complete overlap of (**b,c**) confirms their perfect alignment, and the FZP corresponds to a wavelength of 632.8 nm and a focal length of 30 cm; (**e**) arrangement of LCoS pixels under the reflective polarizing microscope; (**f**) FZP pattern on the LCoS through an optical microscope; P and A represent the transmissive axes of the polarizer and the analyzer, respectively.

The accuracy of the FZP patterns displayed on the LCoS using Matlab software is confirmed using optical microscopic methods. Figure 2a shows that the FZP pattern corresponding to a wavelength of 632.8 nm and focusing at 30 cm is utilized to input the LCoS for macroscopic observation. The size of the LCoS panel was 0.55 inches diagonally, equivalent to 12.49 mm × 7.12 mm. Given that the SLM of JD8554 is a phase-modulating LCoS, the phase of the incident light through LC is modulated to control the polarization state of the outgoing light. Therefore, the pattern observed on the LCoS cannot be readily apparent by direct eye examination. A linear polarizer was positioned in front of the observer to obtain a clear image, which selectively filters out the intensity of polarized light reflected from the regions that exhibit different phase modulation. Hence, variations in amplitude intensities are shown in Fig. 2a. Comparison of the input FZP pattern (Fig. 2b) with the displayed FZP pattern on the LCoS (Fig. 2c) show that they perfectly match in size and fringe positions, as shown in Fig. 2d. Thus, the pattern information provided in Matlab may be used to accurately regulate the pattern size displayed on the LCoS.

Figure 2e shows the arrangement of observed pixels in a reflective polarizing microscope by using an LCoS device with crossed polarizers. The pixels appear as monochromatic squares. As the scale encompasses approximately five pixels and five black matrix lines, the combined width of one pixel and one black line is estimated to be 6.58 μm. According to the specifications of JD8554, the width of one pixel is ~ 6.4 μm. Thus, each black matrix line may have a width of ~ 0.18 μm. Considering the LCoS active area resolution of 1920 × 1080, the dimensions of the entire active area can be estimated as follows: Long side: 1920 × 6.58 μm = 12.63 mm and short side: 1080 × 6.58 μm = 7.11 mm. Figure 2f shows that the FZP pattern corresponding to a wavelength of 632.8 nm and focused at 30 cm is utilized to input the LCoS for a reflective polarizing microscope. The measured (theoretical) radii of the FZP from the innermost to the outermost rings are 424.14 (435.71 μm), 609.56 (616.18 μm), 736.27 (754.67 μm), 862.71 (871.41 μm), 938.69 (974.27 μm), 1050.31 (1067.26 μm), and 1120.57 (1152.77 μm), respectively. Based on the results, the measured values exhibit an error of approximately 3% compared with the theoretical values. This error value is within an acceptable range, and consequently, the size of the FZP displayed on the LCoS can be inferred to closely match the theoretical values.

**Figure 3:**
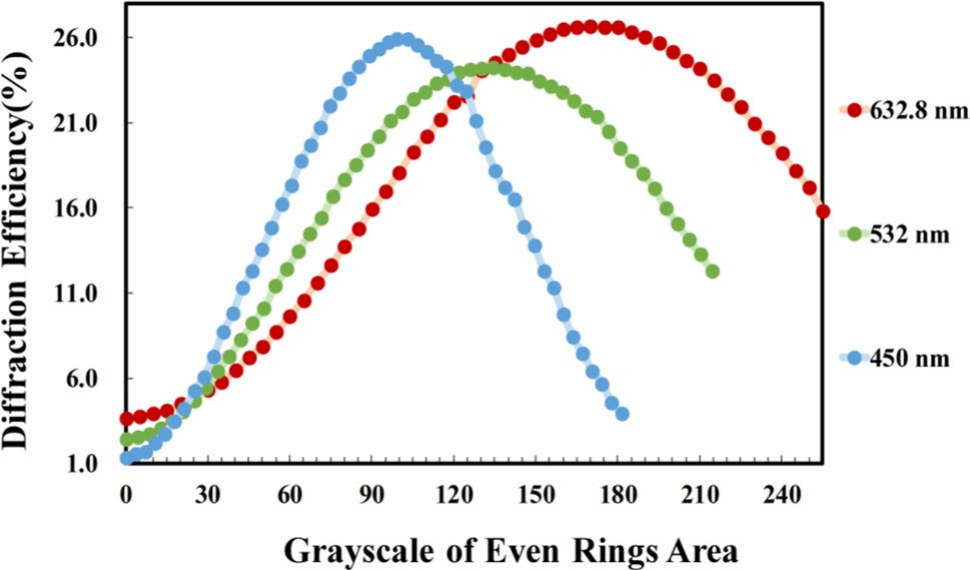
Experimental results of focusing efficiencies of linearly polarized red (R, λ_R_ = 632.8 nm, green (G, λ_G_ = 532 nm, and blue (B, λ_B_ = 450 nm) light sources as functions of the grayscales of even rings.

Figure 3 illustrates the focusing efficiency of linearly polarized monochromatic light sources with different grayscales. We attempt to input signals of FZPs with various grayscales of odd and even rings to obtain the maximum diffraction efficiency. To present FZPs on the RLC-SLM, we must obtain sufficient parameters for the two separate grayscales of the adjacent rings with a phase difference of π. Estimation is simplified by using a focal length of 30 cm to determine the radii of rings comprising different FZPs, and the 0th grayscale is assigned to all odd rings. For optimal focusing efficiency, we determine that even rings with grayscales of 170, 135, and 100 are required for R, G, and B light sources, respectively. The resulting maximum diffraction efficiencies are 26.69% for R, 24.25% for G, and 25.90% for B. Notably, the maximum focusing efficiencies of the three different colors of light sources and focusing points are subsequently lower than the theoretical maximum focusing efficiency (~ 40.5%)^33^. The variations in maximum diffraction efficiency can be caused by human error when the optical setup needs to be adjusted each time the light source is switched. The theoretical maximum focusing efficiency can be achieved by electrically tuning the wavelength-dependent phase difference between neighboring rings of FZPs as ~ π.

**Figure 4:**
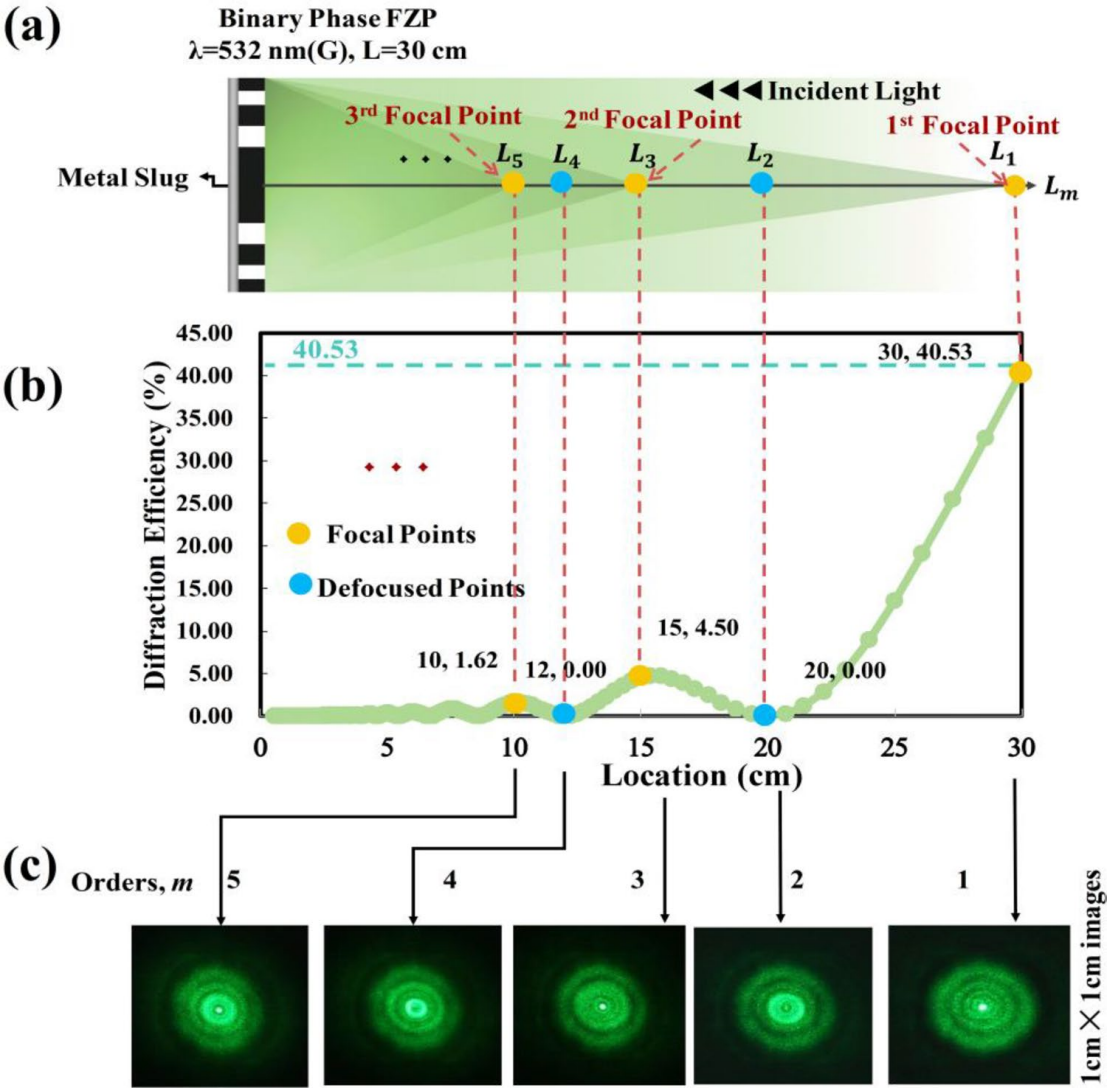
(**a**) Theoretical *m*th-order diffraction efficiencies and focal lengths; (**b**) experimental diffraction patterns of a binary phase FZP corresponding to λ = 532 nm and L = 30 cm.

Figure 4a shows the theoretical *m*th-order of diffraction of a binary phase FZP with a focal length (L) of 30 cm and a wavelength (λ) of 532 nm. The diffraction efficiency can be calculated using the sinc^2^(*m*/2) function, derived from the Fourier Transform^21,30^, where *m* represents the diffraction *m*th order. The first-order diffraction efficiency (*m* = 1) reaches approximately 40.5%. When the adjacent zones (odd and even) of the FZP are configured to be transparent with a phase difference of π (λ/2)^31,34^, the first-order diffraction efficiency at the focal point surpasses that of a conventional FZP with opaque adjacent zones. These high-efficiency FZPs are known as binary phase FZPs. By appropriately selecting grayscale pixel modulations, a binary phase FZP with an exact phase difference can be generated using LCoS-based RLC-SLM technology. As shown in Fig. 4b, the first- to fifth-order diffractions have focal lengths (diffraction efficiencies) of 30 cm (40.53%), 20 cm (0%), 15 cm (4.5%), 12 cm (0%), and 10 cm (1.62%), respectively. As shown in Fig. 4c, the binary FZP exhibit by an LC-SLM caused five relative experimental diffraction patterns recorded by a digital camera.

**Figure 5:**
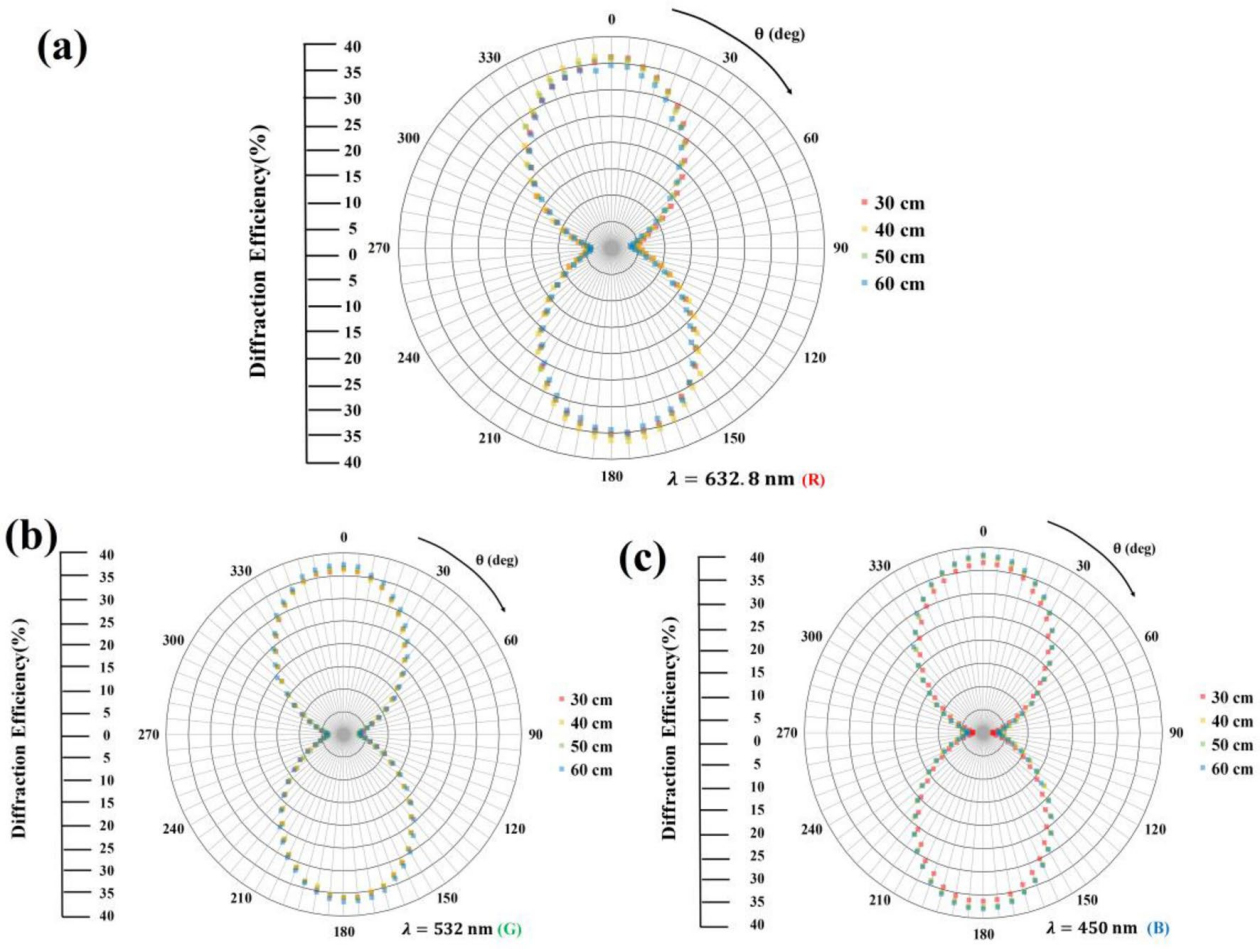
Measured diffraction efficiencies at primary focal points of binary phase-type FZPs using three monochromatic laser lights: (**a**) red (λ_R_ = 632.8 nm); (**b**) green (λ_G_ = 532 nm); and (**c**) blue (λ_B_ = 450 nm).

Figure 5a–c shows the diffraction efficiencies at the primary focal points of binary phase-type FZPs using three monochromatic laser lights with R = 632.8, G = 532, and B = 450 nm as light sources based on LC-SLM. The binary phase-type FZPs are focused at 30, 40, 50, and 60 cm, and the proper grayscales have been set. The diffraction efficiency of the first focus points is determined as Eq. (1):

1 $$\text{Diffraction efficiency} = \frac{\text{Intensity of major focal point}}{\text{Total intensity of reflective light from LCoS}}\times \text{100\%,}$$

when *θ* is between 0° and 180° (i.e., the polarization of the incident light is parallel to the rotating direction of the vertically aligned nematic LCs in LCoS), the LCs exhibit uniaxial optical symmetry with two major refractive indices, namely, effective refractive index $n_{eff}$ and ordinary refractive index $n_{o}$. As such, the LCs have birefringence $\Delta n = n_{eff} - n_{o}$. Consequently, binary phase-type FZPs have maximum diffraction efficiencies of 36%–38%, which are near the theoretical limit value of 40.5%. By contrast, when* θ* is 90° and 270° (i.e., the polarization of incident light is orthogonal to the rotating direction of the vertically aligned nematic LCs in LCoS), the LCs exhibit uniaxial optical symmetry with only one principal refractive index, ordinary refractive index ($n_{o}$), and thus the LCs have no birefringence. Consequently, binary phase-type FZPs have minimum diffraction efficiencies of 2–4%. These correction results demonstrate that the binary phase-type FZP fabricated using LCoS achieves a first-order diffraction efficiency that is quite close to the theoretically inferred value of 40.5%.

**Figure 6:**
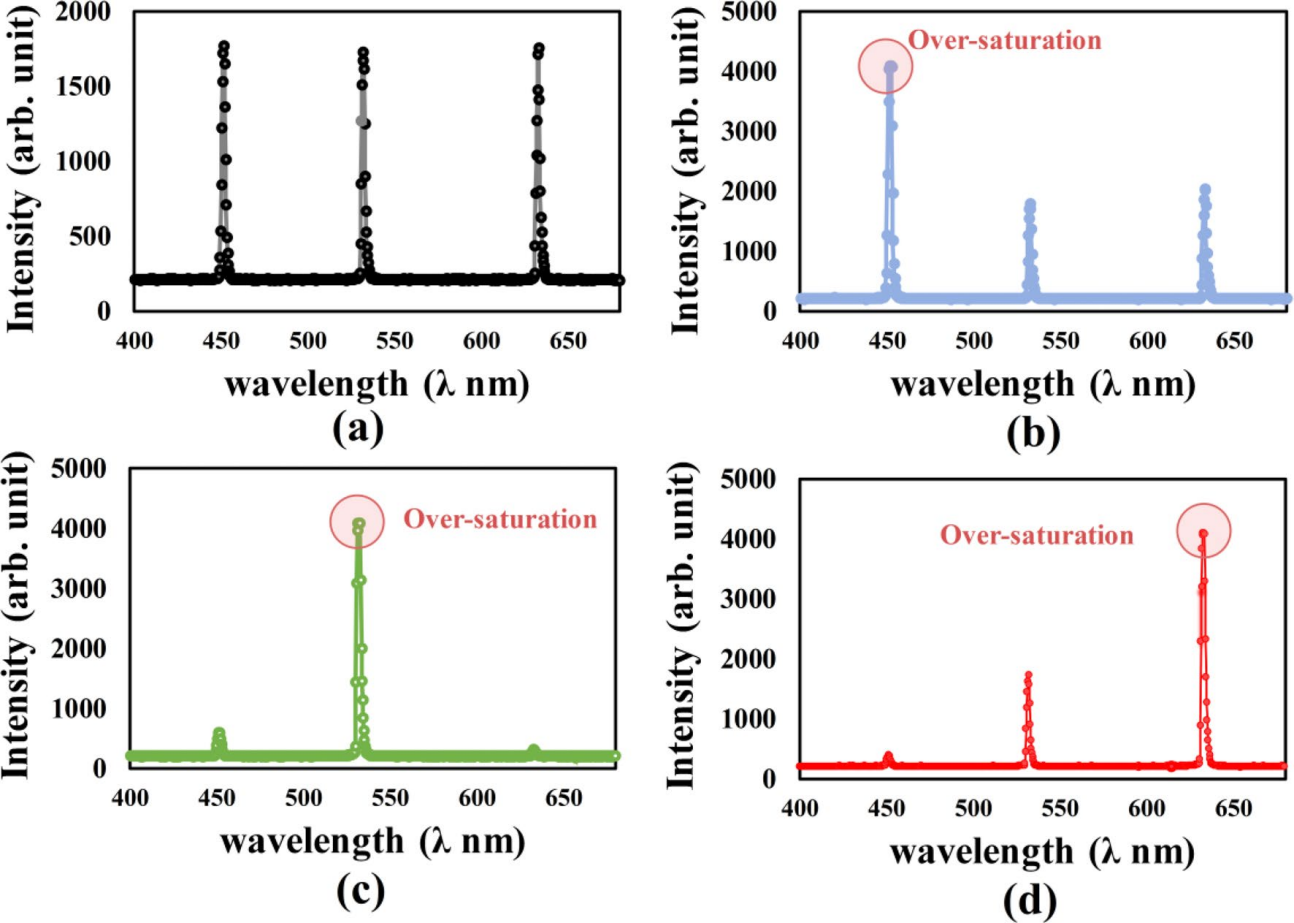
Color separation through FZP-based on RLC-SLM: (**a**) spectra of light sources, mixing with the light of red (λ_R_ = 632.8 nm), green (λ_G_ = 532 nm), and blue (λ_B_ = 450 nm); (**b**) spectra of the FZP to red (λ_R_ = 632.8 nm), (**c**) green (λ_G_ = 532 nm), and (**d**) blue (λ_B_ = 450 nm) light sources at the same primary focal point.

We accurately quantify FZP based on RLC-SLM to precisely measure white-light color separation, as shown in Fig. 6. Figure 6a shows the white light spectrum of an RLC-SLM-based FZP, which uses laser wavelengths of 632.8, 532, and 450 nm. The spectrum is measured at various wavelengths and focal points. Figure 6b–d show the FZP spectra for these three light sources at the main focal point. This experiment demonstrates that the FZP patterns can be focused with the primary light source. By contrast, the other light sources remain out of focus or defocused, resulting in much-decreased intensity for the secondary wavelengths. As a result, the designated FZP maintains defocused energy from other light sources, leading to certain effects. Thus, the FZP pattern focuses on the primary light sources while offering partial or defocused results for other light sources. Hence, the FZP based on RLC-SLM has potential candidates for color separation.

The color modulation effect of the prepared FZP was further examined under mono and polychromatic light source, as shown in Table 1. When the monochromatic light is illuminated in the FZP, the corresponding light source is exhibited at the focal point, while other light sources are unfocused or defocused. By comparison, when dual light source is illuminated in the FZP, a color modulation effect starts to appear. However, the center of the bright spot remains the same color as the initially illumined light source. Further modulation of different colors can be achieved by adjusting the brightness of different light sources or selecting different grayscale levels for the FZP, resulting in varying diffraction intensities and the appearance of other colors. Specifically, although several wavelengths of the polychromatic light do not match the wavelength set by the FZP, the actual result is very close to defocusing situations that resemble focusing.

**Table 1:** Experimental image of focus (⋆) and out of focus (⋄) when the FZP is set for λ_R_ = 632.8 nm and focus on 30 cm. R, G, and B represent the input sources of 632.8 nm, 532 nm, and 450 nm, respectively. These photos are 5 cm × 5 cm.

Source(s)	The illusions of color modulation at 1st focal point		
Single source			
Double sources			
Triple sources			

The following Equation, $R_{n,L} = \sqrt {n\lambda L}$, where *n* is the number of fringes, *L* is the focal length, and the radii corresponding to *n* and *L*, can be used to comprehend this phenomenon further. Equation (2) is used to obtain the relative number of rings *n* and associated three wavelengths^35^:

2 $$\begin{aligned} R_{1,L} = & \sqrt {1 \cdot \lambda \cdot L} \\ = & \sqrt {\frac{{\lambda_{R} }}{{\lambda_{G} }} \cdot \lambda_{G} \cdot L} \, = \sqrt {\frac{632.8}{{532}} \cdot \lambda_{G} \cdot L} \, = \sqrt {1.19 \cdot \lambda_{G} \cdot L} \\ = & \sqrt {\frac{{\lambda_{R} }}{{\lambda_{B} }} \cdot \lambda_{B} \cdot L} \, = \sqrt {\frac{632.8}{{450}} \cdot \lambda_{B} \cdot L} \, = \sqrt {1.41 \cdot \lambda_{B} \cdot L} . \\ \end{aligned}$$

According to Eq. (2), if *n* is a positive integer, the light can travel through the FZP and contribute an optical path difference ${1 \mathord{\left/ {\vphantom {1 2}} \right. \kern-0pt} 2}n\lambda$ at the focal point establishing entirely constructive interference, resulting in a perfect focal point. However, if $n = k - {1 \mathord{\left/ {\vphantom {1 2}} \right. \kern-0pt} 2}$ and k are positive integers, then the optical path difference $n = {1 \mathord{\left/ {\vphantom {1 2}} \right. \kern-0pt} 2}(k - {1 \mathord{\left/ {\vphantom {1 2}} \right. \kern-0pt} 2})\lambda$ at the focal point caused by the light passing through the FZP results in an entirely destructive interference, which results in a defocusing point. According to Eq. (2), if the FRZ light source is set to λ_R_ = 632.8 nm with a focus of 30 cm, then the corresponding value of *n* = 1 indicates that the image has an ideal focal point; if the source is λ_G_ = 532 nm, then the corresponding value is n = 1.19, which is close to 1 and indicates that the image is nearly out of focusing; if the source is λ_B_ = 450 nm, then the corresponding value is n = 1.41, which is close to 1.5 indicates that the image is out of focus or defocusing. Moreover, Eq. (3) calculates the primary focal points of three monochromatic lights, *L*_*R*_, *L*_*G*_, and *L*_*B,*_ for 30.0, 35.68, and 42.19 cm, respectively.

3 $$\sqrt {n \cdot \lambda_{R} \cdot {{L}}_{R} } = \sqrt {n \cdot \lambda_{G} \cdot {{L}}_{G} } = \sqrt {n \cdot \lambda_{B} \cdot {{L}}_{B} } \Rightarrow \left\{ {\begin{array}{*{20}c} {{{L}}_{R} = 30\;{\text{cm }}} \\ {{{L}}_{G} = \frac{{\lambda_{R} }}{{\lambda_{G} }} \cdot {{L}}_{R} = 35.68\;{\text{cm}}} \\ {{{L}}_{B} = \frac{{\lambda_{R} }}{{\lambda_{B} }} \cdot {{L}}_{R} = 42.19\;{\text{cm}}} \\ \end{array} } \right\}.$$

**Figure 7:**
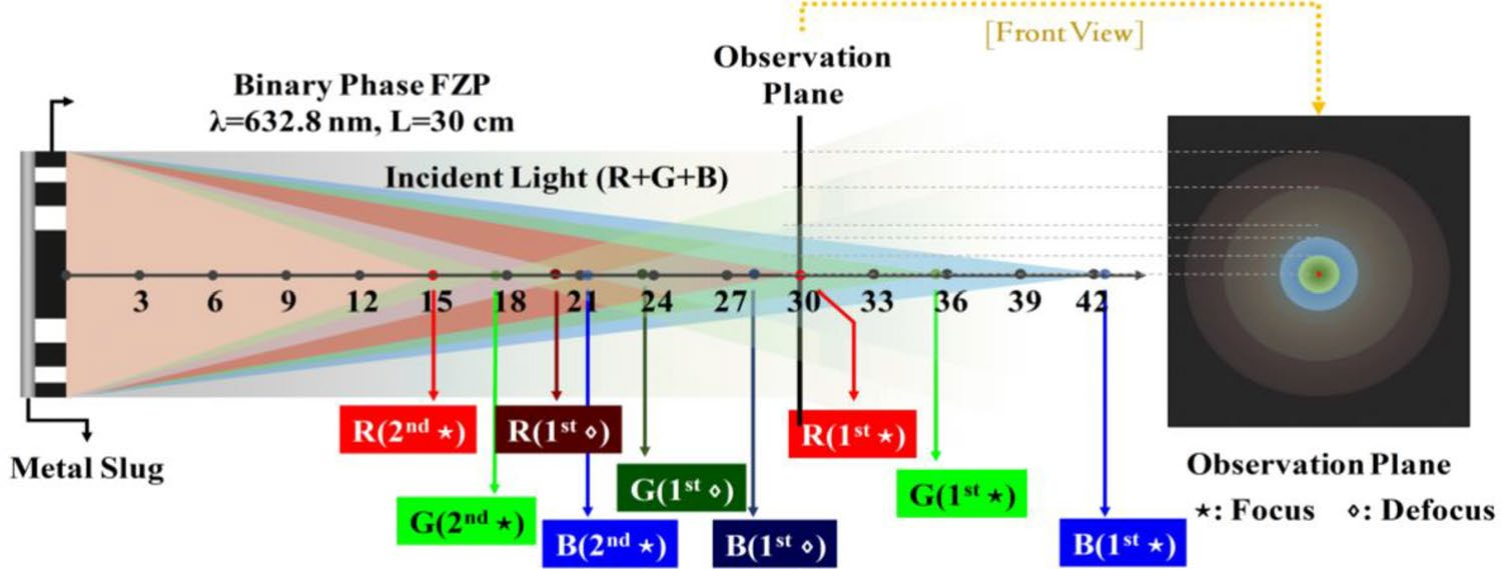
The image at the primary focal point can be plotted according to the calculated results when the FZP is set for λ_R_ = 632.8 nm, focus is on 30 cm, and the light source is mixed with three monochromatic lights of red (λ_R_ = 632.8 nm), green (λ_G_ = 532 nm), and blue (λ_B_ = 450 nm). The simulated result almost conforms to the experimental result in Table 1 of triple sources.

Figure 7 shows the simulation results of the primary focal points of a first-order dark spot and a second-order bright spot for the corresponding FZP under three light sources. Equation (2) discusses the relationship of the refractive index (*n*) and determines the positions, as shown in Table 2. According to Table 2, the imaging color sequence of the FZP three-color input light sources almost matches the expected sequence of color variation, where the focal point is a small red spot in the center.

**Table 2:** Positions of the imaging focus caused by different input light sources for the FZP with a fixed corresponding wavelength of λ = 632.8 nm (focal length of 30 cm).

Light source	1st focal point (cm)	1st defocus point (cm)	2nd focal point (cm)
R	30.0	20.0	15.0
G	35.68	23.79	17.84
B	42.19	28.12	21.09

R, G, and B represent the input light sources at 632.8 nm, 532 nm, and 450 nm, respectively.

We aim to achieve rapid switching of the input white light source by using the corresponding wavelength of the FZP. Rapid color changes at the focus point arise from additive color mixing and switching frequencies faster than the human eye can perceive, which causes white light to mimic a sequential color display. However, the LCoS response limit is 30 ms, which is slower than the human eye limit of 60 Hz. The use of a high-performance RLC-SLM can be highly beneficial to overcome the above limitation. Subsequently, we rapidly switch between different peak wavelengths of FZPs and used a camera’s long exposure of two seconds to simulate the human eye’s inability to discern the switching rate. Table 3 shows the experimental results of color modulation by using corresponding reflective FZPs based on RLC-SLM. As discussed in Fig. 6, when the FZP pattern on the input light source exclusively focuses on that specific light source, other light sources display partial or defocused focusing. As a result, the focal point must match the hue of each wavelength while quickly switching between different related FZPs. For example, when a monochromatic light source is illuminated, the focal point becomes a monochromatic focus. By contrast, when more than one light source is illuminated, the focus point exhibits the modulation result of various overlaid colors. We can obtain (filter out) a desired focal point color by using reflective Fresnel lenses.

**Table 3:** Experimental results of color modulation using corresponding reflective FZPs based on RLC-SLM.

Source(s)	Modulation at 1st focal point		
Single source			
Double sources			
Triple sources			

R, G, and B represent 632.8 nm, 532 nm, and 450 nm input sources. These photos are 5 cm × 5 cm.

## Conclusion

In summary, we have examined adaptive focal length in white light focusing using an RLC-SLM based on an LCoS device. The RLC-SLM device can show various excellent broadband focusing and wavelengths, and can even adjust diffraction efficiencies by modifying the FZPs grayscales. The prepared binary phase-type FZPs experimentally obtained maximum diffraction efficiencies of approximately 38%, close to the theoretical limit of 40.5%. In addition, we use FZPs based on the RLC-SLM device to separate the broadband light source from the desired color source at their focal points. These experimental results demonstrate that the FZPs patterns can focus the light source with the desired wavelength in contrast to other approaches that were out of focus or defocused. Additionally, using real-time switches of FZPs with the appropriate parameters allows for the feasibility to achieve the desired result of white light focusing based on color sequential and additive color approaches. The high agreement between the broadband focusing simulations and experimentally measured results reveal the high processing quality of the FZPs. Overall, the proposed RLC-SLM design paws the way toward a new class for future displays, adaptive camera systems, optical communications, microscopes, holography, portable/wearable devices, and beyond applications.

## Data Availability

The data in this study are not publicly available; however, the data can be provided by the authors upon reasonable request.
